# Supplementary material for: Dietary Patterns and Fibre Intake Are Associated with Disease Activity in Australian Adults with Inflammatory Bowel Disease: An Exploratory Dietary Pattern Analysis
Source: Nutrients. 2024 Dec 17;16(24):4349. doi: 10.3390/nu16244349 (PMC11677955; doi:10.3390/nu16244349)
Supplement: Supplementary file 1 [file nutrients-16-04349-s001.zip › nutrients-3349391-supplementary.pdf]

Table S1. Details on the aggregation of FFQ food items into categories suitable for PCA.

Aggregated Food Groups (n = 32)	Original food Items (n = 144)
Olive Oil	‘Olive oil’, ‘Olive oil on bread’, ‘Olive oil on vegetables’ and ‘Olive oil marg’
Other oils or margarine	‘Canola oil’, ‘vegetable oil’, ‘Canola marg’, ‘Cholesterol lowering marg’, ‘Polyunsaturated marg’ and ‘Margarine on vegetables’.
Butter	‘Butter’, ‘Butter/marg blends’ and ‘Butter/dairy blend on vegetables’
Full-cream milk	‘Full cream milk’ and ‘Flavoured milk’
Low-fat milk	‘Reduced fat milk’ and ‘Skim milk’
Plant-based milk	‘Soy milk’ and ‘Rice milk’
Diet soft drink/water	‘Diet soft drink’ and ‘water’
Sugary beverages	‘Soft drink’ and ‘Fruit juice’ ‘
High-fibre cereals	‘Hi fibre white bread’, ‘Wholemeal Bread’, ‘Multi-grain bread’, ‘Rye Bread’, ‘Soy and linseed bread’, ‘Wheat biscuits / flakes’, ‘Bran cereals’, ‘Mixed grain cereals’, ‘Muesli untoasted’, ‘Muesli toasted’, ‘Porridge average’ and ‘Wholemeal crackers or dry biscuits’
Low-fibre cereals	‘White bread’, ‘Gluten free bread’, ‘Sugary cereals’, ‘Cornflakes’, Nutri - Grain’, ‘Rice cereals’, ‘Special K’, ‘Rice’, ‘Pasta or noodles’, ‘Crackers or dry biscuits not wholemeal’ and ‘Corn chips etc’
Sweet biscuits/cakes/pastries	‘Sweet biscuits’ and ‘Cakes or sweet pastries’
Sauces/Condiments	‘Low calorie, low fat salad dressing’, ‘Mayonnaise’, ‘Oil and vinegar salad dressing’, ‘Tomato sauce Or ketchup’ and ‘Vegemite etc’.
Cheese/Sourcream	‘Ricotta or cottage cheese’, ‘All other cheeses’, ‘Cream or sour cream’.
Dairy dessert	‘Ice-cream’
Yoghurt	‘Yoghurt’
Red Meat	‘Beef or veal’ and ‘Lamb’.
Poultry	‘Chicken’ and ‘Eggs’

<table>
<tr><td>Processed meats</td><td>‘Sausages’, ‘Processed meats’ and ‘Bacon’.</td></tr>
<tr><td>Fish/seafood</td><td>‘Fried fish’, ‘Steamed fish’, ‘Tinned fish’, and ‘Other seafood’.</td></tr>
<tr><td>All fresh fruit</td><td>This data was further separated into three sub-categories:
<table>
<tr><td>Berries</td><td>‘Berries’ and ‘Strawberries’.</td></tr>
<tr><td>All other fresh fruit</td><td>‘Apricot’, ‘Cherries’, ‘Figs’, ‘Plums’, ‘Mango or paw paw’, ‘Grapes’, ‘Peaches or nectarines’, ‘Apples’, ‘Bananas’, ‘Oranges’, ‘Pears’, ‘Cantaloupe’, ‘Watermelon’, ‘Pineapple’ and ‘Kiwi fruit’</td></tr>
<tr><td>Dried or tinned fruit</td><td>‘Dried apricots’, ‘Other dried fruit’ and ‘Tinned fruit’</td></tr>
</table></td></tr>
<tr><td>Starchy veg</td><td>‘Potato cooked in fat’, ‘Potato cooked without fat’ and ‘Sweet potato’.</td></tr>
<tr><td>All non-starchy veg</td><td>This data was further separated into three sub-categories:
<table>
<tr><td>Green veg</td><td>‘Iceburg lettuce’, ‘Other lettuce’, ‘Asian greens’, ‘Other cooked leafy veg’, ‘Brussel sprouts’, ‘Broccoli’, ‘Asparagus’, ‘Celery’, ‘Green beans’ and ‘Green peas’.</td></tr>
<tr><td>Orange/red veg</td><td>‘Carrots’, ‘Beetroot’, ‘Eggplant’, ‘Pumpkin’, ‘Capsicum’, ‘Fresh tomato’, ‘Canned tomato’ and ‘Tomato products’</td></tr>
<tr><td>All other veg</td><td>‘Sweet corn’, ‘Garlic’, ‘Zucchini or squash’, ‘Mushrooms’, ‘Cucumber’, ‘Onion or leek’, ‘Olives’ and ‘Avocado’.</td></tr>
</table></td></tr>
<tr><td>Legumes</td><td>‘Baked beans’ and ‘Dried beans, dried peas, chick peas’</td></tr>
<tr><td>Pizza and pastries</td><td>‘Pizza’, ‘Pastries with cheese’, ‘Pastries with meat’</td></tr>
</table>

Choc/lollies	‘Chocolate’, ‘Other confectionary’, ‘Jam’ and ‘Sugar’.
Nuts	‘Peanuts, peanut butter’ and ‘Other nuts’.
Tea/Coffee	‘Tea’, ‘Herbal tea’, ‘Coffee’ and ‘Coffee substitutes’
Alcohol	‘Heavy beer’, ‘Red wine’, ‘White wine’, ‘SpiritsPremix’ and ‘Spirits’.

Table S2. Australian Dietary Guidelines classification of food groups included in this study.

**5 Food Group (from the Australian Guide to Healthy Eating)**		**Australian Dietary Guidelines classification**		**Examples of foods included in this category**
Code	Name	**Code**	**Name**	
10	Grains (cereal) foods	101	Wholegrain or higher fibre cereal/grains	Wholemeal or mixed-grain bread, cooked brown rice, wholemeal pasta, buckwheat, semolina, popcorn, cooked whole oats.
		102	Refined or lower fibre cereal/grains.	White bread, white rice, white wheat pasta, corn and rice flakes.
20	Vegetables and legumes/beans	201	Green and brassica vegetables	Broccoli, brussels sprouts, cabbage, Chinese cabbage, cauliflower, kale, peas, beans,
		202	Orange vegetables	Carrot, pumpkin
		203	Starchy vegetables	Potato, white & orange sweet potato, taro, cassava, sweetcorn, parsnip
		204	Legumes as vegetables	Cooked dried or canned beans, chickpeas, split-peas, lentils
		205	Other vegetables	Beetroot, celery, sprouts, tomato, , zucchini, avocado, capsicum, cucumber, eggplant, mushroom, onion/shallots, ginger, garlic,
30	Fruit	301	Fresh/canned fruit	Whole, sliced or diced fresh fruit, frozen fruit and canned fruit
		302	Dried fruit	Dried apricots, dried fruit salad, dried dates, sultanas
		303	Fruit juice	100% juice

40	Milk, yoghurt, cheese and/or alternatives	401	Higher fat dairy foods	Feta, parmesan, camembert, brie, cheddar, regular fat cream cheese, haloumi, paneer, regular fat milk powder used in recipes.
		402	Medium fat dairy foods	Regular fat/full cream milk, ricotta and cottage cheese, reduced fat processed cheddar cheese, Greek style yoghurt, regular fat soy and cereal based beverage.
		403	Lower fat dairy foods	Reduced fat or skim milk, low fat pr
50	Lean meats and poultry, fish, eggs, tofu, nuts and seeds and legumes/beans	501	Red meat, lean (<10% fat)	Cooked lean red meats such as beef, lamb, veal, pork, sausages with a fat content <10%, Lean leg ham, trimmed bacon
		502	Red meat, non-lean (≥10% fat)	Cooked beef, lamb, mutton and pork with fat content ≥10%, Regular sausages, non-lean processed meats such as untrimmed bacon, prosciutto, pastrami, salami and chorizo.
		503	Poultry, lean (<10% fat)	Cooked chicken, turkey and duck with fat content <10%, roast turkey deli slice, processed turkey luncheon meat
		504	Poultry, non-lean (≥10% fat)	Chicken, turkey and duck with fat content ≥10% fat, regular fat processed chicken luncheon meat
		505	Fish and seafood	Includes both fresh and canned fish, prawns, crab, lobster, mussels, oysters, scallops and clams.
		506	Eggs	Whole eggs, egg yolks, egg whites
		507	Legumes as meat alternative	Baked beans, canned beans, cooked dried beans, lentils, chick peas, split peas and vegetable patties (usually lentil/chickpea based)
		508	Nuts and seeds	Nuts, seeds, nut butters, tahini, nut/seed pastes, no added salt
60	Water	Includes domestic water (tap, tank/rain water) and purchased packaged water including mineral water		
70	Unsaturated spreads and oils	701	Unsaturated spreads	Polyunsaturated and monounsaturated margarines, olive oil spreads
		702	Unsaturated oils	Polyunsaturated and monounsaturated oils

		703	Nuts, nut pastes and seeds	Tree nuts, peanuts, nut pastes/butters, seeds and seed pastes (e.g. tahini)

Table S3. Classification of fermented food and drink categories

**Fermented food category**	**Items included**
Fermented dairy/dairy alternatives	Yoghurt, labneh and kefir were identified in this category. Additionally, all fresh or frozen yoghurt products including frozen yoghurt, yoghurt dressing and smoothies with yoghurt were classified as a fermented product.
Fermented non-dairy drinks	Kombucha and items labelled as “fermented” for example fermented cola drink, were included. Apple Cider Vinegar was classified as a fermented non-dairy drink, provided it was recorded as a brand name unfiltered and therefore containing “the mother”.
Fermented vegetables	Sauerkraut, kimchi, tempeh and miso
Probiotic supplement/food product	If the product name included the word probiotic, it was considered in this category. For example, probiotic yoghurt and probiotic kefir. Any product with added probiotics was considered a probiotic product, and not a duplicate of another category. For example, probiotic yoghurt was not counted in the fermented dairy category.

Methods S1: Details of steps undertaken to merge food group and Fibre Categories Database data with the 3-day food record.

The following data management steps were undertaken to enable merging with food group and fibre data:

1. AUSNUT ID, available from the AUSNUT 2011-13 AHS Food Details File from the FSANZ website, was matched to the appropriate FoodWorks FoodName based on the corresponding FoodID from FoodWorks. Majority of this matched coding was provided by Xyris. For FoodNames not automatically assigned an AUSNUT ID by Xyris, the researcher (DC) manually assigned this by choosing the AUSNUT ID of the most similar corresponding food item in the AUSNUT Database. For example, coconut yoghurt items were assigned the AUSNUT ID of Soy yoghurt and oat milk assigned the AUSNUT ID of Soy milk.
2. The FoodID from the Australian Health Survey - Australian Dietary Guidelines Database, available at FSANZ website was then matched to this AUSNUT ID. The corresponding Australian Dietary Guidelines food group categories matched to the FoodID were merged with the main data file according to the AUSNUT ID.
3. Fibre Categories Database AUSNUT Code, which is the same as the SurveyID from the Australian Health Survey Australian Dietary Guidelines Database, was then merged with the dataset. We renamed this SurveyID to AUSNUT Code for merging and consistency.

Table S4. Disease characteristics amongst IBD patients

Characteristic	CD (n = 117)	UC (n = 106)	P-value
**Age at diagnosis, median (IQR)**	30 (21-42)	30 (22-39)	0.570†
**Disease duration, median (IQR)**	12 (7.2-21)	12 (5.7-21)	0.675†
**Extra-intestinal manifestation, N (%)**	36/115 (31.3%)	19/104 (18.3%)	0.039*‡
Ulcerative colitis			
**Extent**<br>**- Proctitis**<br>**- Left-sided**<br>**- Extensive**		 - 18/105 (17.1%)<br>- 40/105 (38.1%)<br>- 47/105 (44.8%)	
- **Prior episode of ASUC**		34/99 (34.3%)	
- **Prior colectomy**		5/106 (4.7%)	
Crohn's disease			
**Distribution**<br>**- Ileal**<br>**- Colonic**<br>**- Ileocolonic**<br>**- Upper GI**<br>**- Perianal**	 - 32/117 (27.4%)<br>- 47/117 (40.2%)<br>- 38/117 (32.5%)<br>- 5/117 (4.3%)<br>- 32/117 (27.4%)		
- **Phenotype**<br>**- Non-stricturing, non-penetrating**<br>**- Stricturing**<br>**- Penetrating**	- 56/113 (49.6%)<br>- 41/113 (36.3%)<br>- 16/113 (14.2%)		
- **Abdominal surgery**	40/117 (34.2%)		
***Current medications,* N (%)**			
**Oral and/or topical 5-ASA**	N/A	79/106 (74.5%)	
**Thiopurines**	44/117 (37.6%)	25/106 (23.6%)	0.034*‡
**Methotrexate**	11/117 (9.4%)	2/106 (1.9%)	0.021*§
**Any advanced therapy**<br>**- Anti-TNF**	66/117 (56.4%)<br>- 53/117 (45.3%)	34/106 (32.1%)<br>- 16/106 (15.1%)	<0.001*‡

**- Vedolizumab**<br>**- Ustekinumab**<br>**- Tofacitinib**	- 4/117 (3.4%)<br>- 9/117 (7.7%)<br>- N/A	- 15/106 (14.2%)<br>- 0/106 (0%)<br>- 3/106 (2.8%)	

†p-values from the Mann-Whitney test

‡ p-values from Chi-squared test of between-group differences

§ p value from Fishers exact test of between-group differences

*p-value <0.05, indicating statistical significance

Abbreviations: CD, Crohn's disease; UC, ulcerative colitis; ASUC, acute severe ulcerative colitis; GI, gastrointestinal; 5-ASA, 5-aminosalicylate; TNF, tumour necrosis factor-alpha

Table S5. Nutrient intake amongst the IBD and HC cohorts.

Nutrient	IBD	HC	P-value
**Total energy, kJ/day**	7579 (6411 – 8725)	7504 (6203 – 8825)	0.568
**Protein, g/day**	87.9 (72.8 – 100.0)	86.3 (70.7 – 101.0)	0.814
**Saturated fat, g/day**	24.0 (19.0 – 31.6)	23.9 (17.1 – 30.7)	0.271
**Iodine, μg/day**	130.0 (102.0 – 171.0)	135.0 (103.0 – 168.0)	0.766
**Iron, mg/day**	9.44 (7.62 – 11.6)	9.21 (7.45 – 11.1)	0.521
**Calcium, mg/day**	661.0 (505.0 – 857.0)	707.0 (536.0 – 871.0)	0.356
**Zinc, mg/day**	9.23 (7.65 – 11.3)	9.24 (7.30 – 11.0)	0.643
**Vitamin A, μg/day**	778.0 (545.0 – 1106.0)	812.0 (570.0 – 1047.0)	0.937
**Vitamin B12, μg/day**	3.55 (2.47 – 4.56)	3.60 (2.67 – 4.59)	0.639
**Vitamin C, mg/day**	70.1 (45.8 – 110.0)	74.4 (50.8 – 98.2)	0.883
**Vitamin E, mg/day**	10.5 (7.99 – 13.8)	10.6 (8.39 – 13.2)	0.798

†p-values from the Mann-Whitney test

Figure S1. Image of scree plot of eigenvalues for determination of IBD dietary patterns to retain.

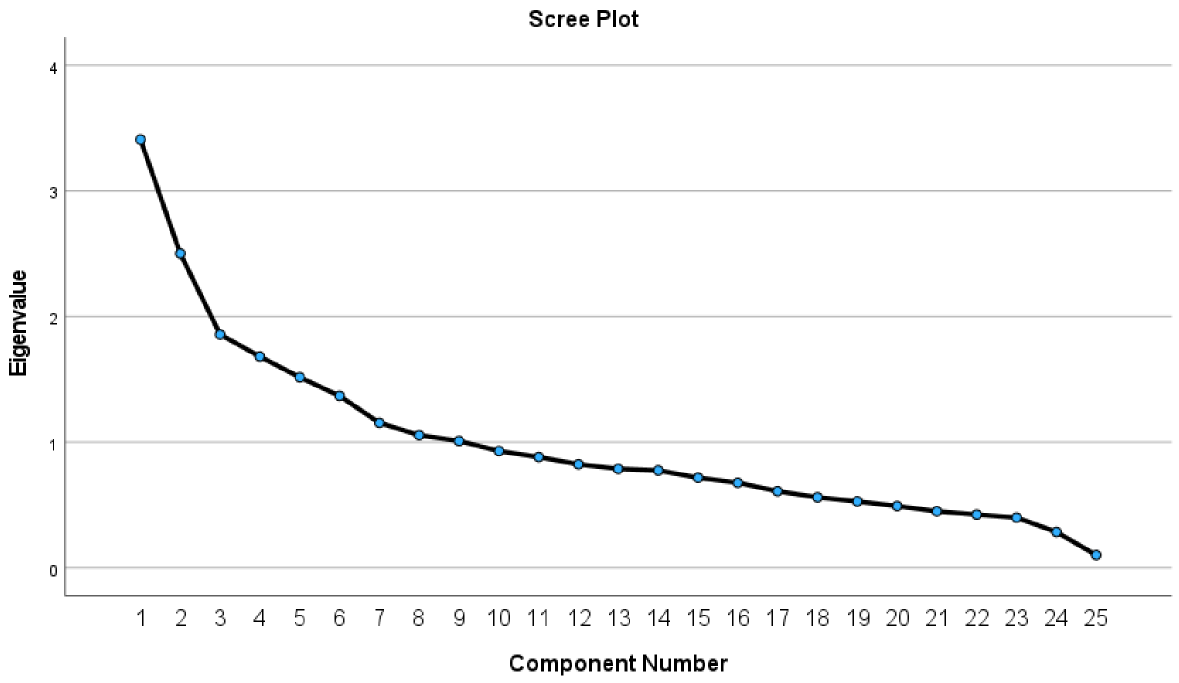

Methods S2. Methods and results for dietary cluster analysis

Determination of dietary patterns using Cluster Analysis

K-means cluster analysis was performed in SPSS on the FFQ data separately for participants with IBD and healthy controls to determine dietary clusters of median food intake in g/day [82, 83]. Cluster analysis groups individuals into mutually exclusive groups according to individual differences in mean dietary intake [84]. Input variables are grouped according to how they correlate, creating distinct patterns of food variables correlated together from the dietary data [85]. K-means cluster solutions of 2-8 clusters were computed, consistent with previous similar studies. Clusters containing <10% of the total sample were not retained as they were considered too small for adequate statistical power [86]. The retained clusters were labelled according to the predominant food groups therein.

Three dietary clusters were identified for adults with IBD (Cluster 1: n = 77; Cluster 2: n = 55; Cluster 3: n = 82). Ten participants were excluded due to missing or incomplete data. Cluster 1, labelled 'Prudent', was characterised by the highest intake of plant-based milk, water or diet soft drink, berries and nuts. Cluster 2, labelled 'Western', was characterised by the highest intake of full-cream milk, low-fibre cereals, sauces and condiments, butter, pork, fresh fruit, tea/coffee and alcohol. Cluster 3, labelled 'Light diet', did not contain the highest intake of any food variables, however, was characterised by the lowest intake of water/diet soft drink, berries, fresh fruit, nuts, tea/coffee and alcoholic beverages (Table S6)

Similarly, three dietary clusters were identified for the HC cohort (Cluster 1: n = 39; Cluster 2: n = 67s; and Cluster 3: n = 76). Seven participants were excluded from the analysis due to missing or incomplete data. Cluster 1, labelled 'Tea, coffee and alcohol', was characterised by the highest intake of tea/coffee, alcoholic beverages and high-fibre cereal. Cluster 2, labelled 'Prudent', was characterised by the highest intake of plant milk, water/diet soft drink, green vegetables, orange/red vegetables and other vegetables. Cluster 3, labelled 'Western', did not contain the highest intake of any food variables, however, was characterised by the lowest intake of water/diet softdrink, berries, green vegetables, other vegetables, tea/coffee and alcoholic beverages (Table S7)

Table S6. Median intake (g/day) for each food variable within the three distinct dietary clusters for the IBD cohort.

**Food variable**	**Prudent** (n= 77, 36.0%)	**Western** (n = 55, 25.7%)	**Light diet** (n = 82, 38.3%)	**p-value †**
Olive oil	12.0	15.3	15.8	0.200
Other oils/margarine	9.7	12.1	10.7	0.471
Full-cream milk	*64.8*‡	**143.8**	119.5	0.20
Low-fat milk	51.2	69.3	103.5	0.077
Plant milk	**94.3**	*25.6*	77.2	0.020
Diet soft drink/water	**2011.0**	1020.0	*903.0*	<0.001
Sugary beverage	233.7	99.2	179.3	0.103
Low-fibre cereals	*107.3*	**145.7**	108.8	0.011
High-fibre cereals	72.5	87.1	82.7	0.607
Chocolate/lollies	19.7	20.6	20.0	0.942
Sweet biscuits/pastries	17.1	21.3	17.5	0.487
Pizza/pastries	21.6	34.2	20.0	0.127
Sauces/condiments	*9.7*	**13.0**	8.5	0.033
Butter	*2.8*	**6.0**	5.4	0.007
Cheese/sourcream	18.0	19.3	18.9	0.900
Dairy dessert	6.5	5.6	5.6	0.815
Yoghurt	35.2	30.5	31.2	0.738
Red meat	55.9	61.8	68.5	0.306
Poultry	93.3	102.4	90.2	0.462
Pork	10.7	**20.5**	*9.6*	0.016
Processed meats	15.6	20.7	18.8	0.284
Seafood	40.5	40.3	37.1	0.827
Berries	**23.5**	16.5	*15.7*	0.044
Other fresh fruit	185.9	**204.0**	*139.7*	0.014
Dried/tinned fruit	11.9	7.7	10.4	0.565
Starchy vegetables	34.3	25.3	34.3	0.124
Green vegetables	68.3	66.0	65.6	0.959
Orange/red vegetables	178.5	171.1	157.5	0.547

Other vegetables	59.1	57.6	50.1	0.343
Legumes	54.1	72.2	49.6	0.336
Nuts	**22.4**	15.8	*15.2*	0.047
Tea/coffee	457.8	**1063.6**	*371.8*	<0.001
Alcoholic beverages	576.8	**1234.7**	*422.3*	<0.001

† p-value tested with ANOVA

‡The highest and lowest median intake for each food variable with a significant difference among clusters are marked bold and italic, respectively

Table S7. Median intake (g/day) for each food variable within the three distinct dietary clusters for the HC cohort.

**Food Variable**	**Tea, coffee and alcohol** (n = 39, 21.4%)	**Prudent** (n = 67, 36.8%)	**Western** (n = 76, 41.8)	**p-value†**
Olive oil	14.2	14.5	14.6	0.984
Other oils/margarine	8.4	8.8	10.0	0.710
Full-cream milk	126.6	89.7	132.7	0.306
Low-fat milk	136.2	65.3	112.3	0.124
Plant milk	*30.4‡*	**85.7**	30.6	0.027
Diet soft drink/water	1046.0	**1832.0**	*686.0*	<0.001
Sugary beverage	67.1	122.1	92.1	0.281
Low-fibre cereals	119.8	135.5	130.0	0.672
High-fibre cereals	**108.3**	*72.2*	96.6	0.077
Chocolate/lollies	14.8	16.4	20.8	0.110
Sweet biscuits/pastries	11.4	13.5	16.1	0.317
Pizza/pastries	23.6	20.1	29.9	0.239
Sauces/condiments	9.6	11.5	8.5	0.150
Butter	4.8	4.3	5.3	0.605
Cheese/sourcream	17.7	17.7	17.3	0.984
Dairy dessert	3.8	5.5	7.7	0.086
Yoghurt	42.2	44.9	47.3	0.883
Red meat	47.2	55.8	66.8	0.085
Poultry	84.9	90.3	93.2	0.689
Pork	14.1	17.1	16.1	0.845
Processed meats	12.2	14.3	14.9	0.599
Seafood	44.3	43.2	36.6	0.261
Berries	23.8	**27.3**	*16.4*	0.033
Other fresh fruit	224.6	212.7	195.8	0.490
Dried/tinned fruit	7.0	6.1	9.9	0.348

Starchy vegetables	27.6	24.0	23.9	0.659
Green vegetables	84.5	**87.0**	*65.6*	0.041
Orange/red vegetables	189.6	**212.6**	*166.0*	0.095
Other vegetables	57.2	**74.3**	*54.2*	0.010
Legumes	71.4	86.9	48.2	0.101
Nuts	20.3	25.6	22.5	0.528
Tea/coffee	**1213.4**	481.0	*468.4*	<0.001
Alcoholic beverages	**1332.0**	594.5	*560.6*	<0.001

†p-value tested with ANOVA

‡The highest and lowest median intake for each food variable with a significant difference among clusters are marked bold and italic, respectively
